# Neuropathic pain, antidepressant drugs, and inflammation: a narrative review

**DOI:** 10.1186/s44158-024-00204-z

**Published:** 2024-09-27

**Authors:** Giulia Catalisano, Gioacchina Martina Campione, Giulia Spurio, Alberto Nicolò Galvano, Cesira Palmeri di Villalba, Antonino Giarratano, Antonietta Alongi, Mariachiara Ippolito, Andrea Cortegiani

**Affiliations:** 1 Department of Precision Medicine in Medical Surgical and Critical Care (Me.Pre.C.C.), University of Palermo, Palermo, Italy; 2 Department of Anesthesia Intensive Care and Emergency, University Hospital Policlinico ‘Paolo Giaccone’, Via del Vespro 129, 90127 Palermo, Italy

**Keywords:** Neuropathic pain, Pain, Antidepressant drugs, Antidepressants, Neuroinflammation, Cytokines

## Abstract

**Background:**

Neuropathic pain (NP) is a chronic and disabling condition, caused by a lesion or disease of the somatosensory nervous system, characterized by a systemic inflammatory state. Signs and associated symptoms are rarely recognized, and response to usual analgesic drugs is poor. Antidepressant drugs are first-line agents for the treatment of NP. This narrative review aims to summarize the role of antidepressant drugs in treating NP and their mechanism of action, focusing on the effects on inflammatory cytokines.

Main text.

Peripheral nerve injury leads to a local inflammatory response and to the disruption of the blood-medullary barrier, allowing the influx of peripheral immune cells into the central nervous system. Antidepressants have antinociceptive effects because they recruit long-term neuronal plasticity. Amitriptyline modulates the inflammatory response due to the reduction of the mRNA of pro-inflammatory cytokines acting as an adenosine agonist and leading to the activation of the A_3_AR receptor. Through toll-like receptors, local inflammation determines the release of pro-inflammatory cytokines such as tumor necrosis factor-alpha (TNF-α) that drives and stimulates the hyperflammation in NP. Nortriptyline has an important antiallodynic effect in NP as it determines the recruitment of norepinephrine in the dorsal root ganglia. By modulating the β2-adrenoreceptors expressed by non-neuronal satellite cells, it inhibits the local production of TNF-α and determines a reduction of NP symptoms. Following the administration of antidepressants, there is a reduction in the production of TNF-α in the brain which in turn transforms the function of the α2-adrenergic receptor from an inhibitor to an activator of the release of norepinephrine. This is important to prevent the development of chronic pain.

**Conclusion:**

Inflammatory cytokines are the main players in a bidirectional communication between the central and peripheral nervous system and the immune system in NP. Antidepressants have an important role in NP. Further research should explore the interaction between neuroinflammation in NP, the effects of antidepressants and the clinical relevance of this interaction.

## Introduction

The history of pain research is long, but only after the foundation of the International Association for the Study of Pain (IASP), the attention was focused on neuropathic pain (NP) and its treatment [1]. Evolutionarily, pain develops to protect the individual from potential harm [2]. However, not all types of pain play an adaptive and protective role [2]. Therefore, knowing the etiology and pathophysiology of NP is fundamental to initiate correct pain modulation. Specifically, according to the IASP guidelines for the treatment of NP, tricyclic antidepressants, selective serotonin and norepinephrine reuptake inhibitors, pregabalin, and gabapentin are recommended as systemic first-line treatments [3]. Different studies [4] – [9] have demonstrated changes in cellular activity and the development of an inflammatory state in the case of NP [10]. In particular, it has been shown that the upregulation of autophagic activities can directly alleviate NP [10]. This process is regulated by pro-inflammatory cytokines, in particular tumor necrosis factor-(TNF-α) and interleukin 6 (IL-6) [9]. TNF-α drives the hyperinflammation and the synthesis of other pro-inflammatory cytokines, involved in pain-related pathways, also inducing and modulating NP [9]. Through this mechanism, TNF-α regulates the balance between cell survival and death, and at the same time, it induces and modulates NP [9]. Therefore, therapies that stimulate autophagic activities or suppress pro-inflammatory cytokines are useful for treating NP [10]). This narrative review aims to summarize the role of antidepressant drugs in treating NP and their mechanism of action, focusing on the effects on inflammatory cytokines.

## Methods

For the purpose of this narrative review, a systematic search was performed on the PubMed database to survey the most relevant literature on NP and the role of antidepressant drugs. The search included the following keywords: “Neuropathic pain” or “Neuropathic pain syndrome” and “Antidepressant drugs” as exact phrases and as a combination of broad subject headings according to database syntax. The search was restricted to the last 20 years. Relevant records were narratively summarized by the authors.

## Definitions, classification, and epidemiology

According to IASP, pain is defined as an unpleasant sensory and emotional experience associated with, or resembling that associated with, actual or potential tissue damage [11]. Nociceptive, nociplastic, and neuropathic pain are the three branches used to describe different types of pain. IASP defines nociceptive pain as pain that arises from actual or threatened damage to non-neural tissue and is due to the activation of nociceptors. The nociceptive pain results from the activation of nociceptive sensory axon by noxious stimuli [12]. Generally, it is finite, localized, and subside with healing or removal of the noxious substance. Nociplastic pain arises from altered nociception despite no clear evidence of actual or threatened tissue damage causing the activation of peripheral nociceptors or evidence for disease or lesion of the somatosensory system causing the pain [11]. Examples are pain due to fibromyalgia or migraine headaches. According to IASP’s latest definition, Neuropathic pain is caused by a lesion or disease of the somatosensory nervous system [11]. NP is often a chronic and disabling condition that is poorly responsive to usual analgesic drugs [12].

Based on etiology, NP can be classified as either central or peripheral [13]. The most relevant causes of chronic peripheral NP are trigeminal neuralgia, peripheral nerve injury, diabetic peripheral neuropathy, postherpetic neuralgia, HIV, and chemotherapy, as well as trauma or surgical procedures. Instead, spinal cord injury, brain injury, post-stroke, or multiple sclerosis are the cause of chronic central NP [12].

NP affects about 7–10% of the world’s population and in particular patients over 50 years of age [12]. Women show a higher prevalence of NP than men [14].

## Symptoms and diagnosis

Patients affected by NP develop a wide range of symptoms that can be positive, such as paresthesia or hyperalgesia, or negative (i.e., sensory less) [2]. However, symptomatic manifestations also depend on the cause and site of the nerve lesion [12]. Pain may be perceived as persistent or intermittent spontaneous pain. In addition to pain, patients with NP experience other symptoms including burning, tingling, numbness, and allodynia [13]. These may often worsen patients’ quality of life, both physically and emotionally [15]. Patients may develop refractory pain, difficult to manage [16], and experience anxiety, depression, sleep disorders, and even suicide attempts [17]. Signs and symptoms of NP are often not recognized, resulting in a lack of diagnosis or misdiagnosis [17]. So, to avoid improper diagnosis, physicians should seek a detailed medical history and carry on a complete medical and neurological examination defining the timing of onset, location, and distribution of the pain and its possible association with trauma and sensory deficits [1]. In addition, the quality of pain should be recorded using pain descriptors, i.e., shooting, burning, or aching, provided by the patient [1]. Pain intensity can be quantified using a numeric rating scale (NRS) or alternatively with the visual analogue scale [18]. Questionnaires have been developed to help physicians identify NP symptoms. They are easy to use, exist in more than 90 languages​, and are validated for patients with pain exclusively or predominantly in a single site of the body [19]). The most used are the Leeds Assessment of Neuropathic Symptoms and Signs (LANSS), the Douleur Neuropathique en 4 Questions (DN4), their self-administered versions (S-LANSS and I-DN4, respectively), and the PainDETECT [19]. The extent of the neuropathic component of chronic pain syndromes can be assessed with the Neuropathic Pain Symptom Inventory (NPSI) and the Neuropathic Pain Scale (NPS) (Schlereth and Schlereth [18]). Questionnaires can provide a good overview of the subjective perception of pain and the psychosocial component of pain as a supplement to clinical examination [18].

**Fig. 1:**
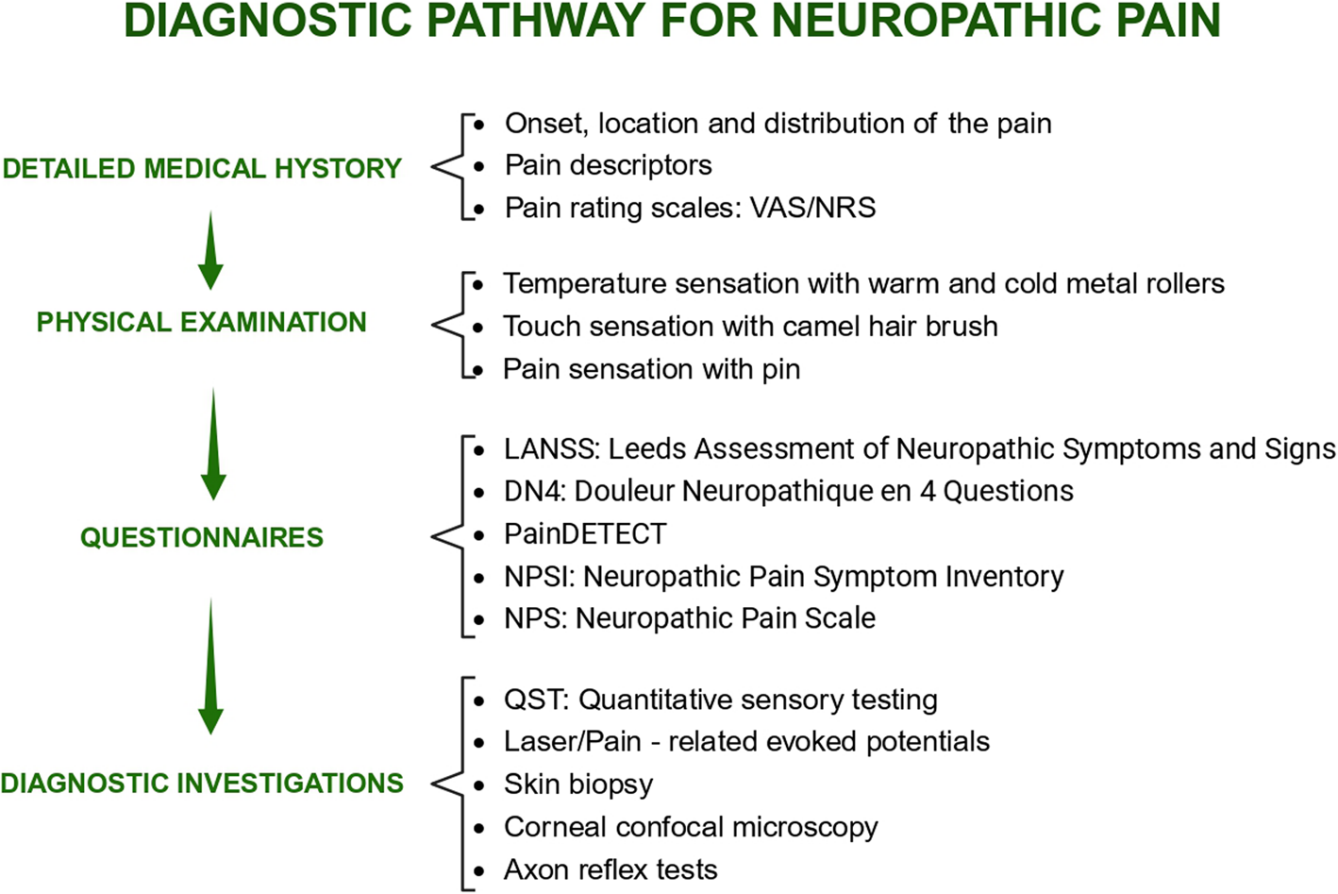
Diagnostic pathway for neuropathic pain. At first, a detailed medical history is useful to recognize signs and symptoms of NP. Timing of onset, location, and distribution of the pain and its possible association with trauma are analyzed. In addition, the quality of pain should be recorded using pain descriptors, i.e., shooting, burning, or aching. Pain intensity can be quantified using a numeric rating scale (NRS) or alternatively with the visual analogue scale. Afterwards, a physical examination is necessary to evaluate temperature sensation with warm and cold metal rollers, touch sensation with camel hairbrush, and pain sensation with pins. Subsequently, questionnaires are offered to patients to provide a good overview of the subjective perception and the psychosocial component of pain. The most used are the Leeds Assessment of Neuropathic Symptoms and Signs (LANSS), the Douleur Neuropathique en 4 Questions (DN4), their self-administered versions (S-LANSS and I-DN4 respectively), the PainDETECT, the Neuropathic Pain Symptom Inventory (NPSI), and the Neuropathic Pain Scale (NPS). Lastly, to establish the presence of lesions or diseases of the somatosensory system, diagnostic investigations are employed. These include quantitative sensory testing (QST), neurophysiological testing with laser/pain-related evoked potentials, skin biopsy, corneal confocal microscopy, and axon reflex tests. QST uses standardized mechanical and thermal stimuli to examine nociceptive and non-nociceptive afferent pathways in peripheral nerves and the central nervous system. Nerve conduction studies and the use of somatosensory evoked potentials are useful to demonstrate, localize, and quantify damage along peripheral or central sensory pathways. Among the neurophysiological techniques, the most used are the evoked potentials of nociceptive stimuli: they can be radiant or contact thermal stimuli, which selectively activate nociceptors, giving rise, respectively, to potentials evoked by laser and contact heat. In case of inconclusive electrophysiological tests or when small fiber pathology is suspected, skin biopsy may be used to confirm a somatosensory lesion. Skin biopsy is a minimally invasive procedure in which a few millimeters of skin containing intraepidermal C fibers are obtained, which are then stained immunohistochemically

According to IASP, NP is a clinical description that requires a demonstrable lesion or a disease that satisfies established neurological diagnostic criteria. To establish the presence of lesions or diseases of the somatosensory system, diagnostic investigations are employed. These include quantitative sensory testing (QST), neurophysiological testing, or skin biopsy [19]. QST uses standardized mechanical and thermal stimuli to examine nociceptive and non-nociceptive afferent pathways in peripheral nerves and the central nervous system [19]. The QST can detect both positive symptoms such as allodynia, paresthesia, mechanical, heat, or cold hyperalgesia, and negative symptoms like loss of mechanical or thermal sensation [18]. Nerve conduction studies and the use of somatosensory evoked potentials are useful to demonstrate, localize, and quantify damage along peripheral or central sensory pathways [19]. Among the neurophysiological techniques, the most used are the evoked potentials of nociceptive stimuli: they can be radiant or contact thermal stimuli, which selectively activate nociceptors, giving rise, respectively, to potentials evoked by laser and contact heat [19]. In case of inconclusive electrophysiological tests or when small fiber pathology is suspected, skin biopsy may be used to confirm a somatosensory lesion [18]. Skin biopsy is a minimally invasive procedure in which a few millimeters of skin containing intraepidermal C fibers are obtained, which are then stained immunohistochemically [18]. The existing tools for diagnosis are summarized in Fig. 1. Some diseases, such as trigeminal neuralgia, are currently defined by their clinical presentation rather than by objective diagnostic testing. Other diagnoses such as postherpetic neuralgia are normally based upon the history [1]. In such instances, clinical judgment is pivotal to elaborate the complexity of symptoms and manifestations and to direct patients towards the highest probable diagnosis and adequate treatment.

## Pathophysiology

The genesis of NP was investigated by Bonezzi et al. finding that the most involved nerve fibers are Aδ and C [13] Acting on the peripheral C fibers would lead to greater pain control and fewer changes at a central level [13]) The transmission of signals occurs through the activation of voltage-gated sodium channels (NaV), which generate and propagate the action potential. By acting on peripheral sodium channels and modulating their function, the propagation of painful stimuli at a central level could be prevented [13]. Thermotransient receptors (TRP) also participate in the modulation of NP: they provide information about thermal changes in the environment and they are expressed at the level of primary sensory nerve terminals [13]. Targeting inflammatory mediators could be useful to control the modulation of thermo-TRPs [13].

Damage to peripheral nerves leads to local inflammatory response [8]. The first cells to react are Schwann cells together with resident immune cells (mast cells and macrophages). Schwann cells dedifferentiate and begin the process of degradation of the myelin sheath at the site of injury. Resident mast cells degranulate, releasing inflammatory mediators, including histamine, serotonin, growth factors, and leukotrienes that sensitize nociceptors and contribute to the recruitment of neutrophils, the first cells to infiltrate damaged tissue [8]. Neutrophils release mediators that sensitize nociceptors and recruit macrophages and T cells to the site of injury. Recruited infiltrating macrophages join resident macrophages and, together with Schwann cells, take part in the phagocytosis of degenerated axons and myelin sheaths. Moreover, they release pro-inflammatory cytokines/chemokines [8]. T-helper cells release pro-inflammatory cytokines such as IL-1b, TNF-α, and IL-17, and also anti-inflammatory cytokines such as IL-4 and IL-10 [8]. These mediators can have a direct effect by binding receptors on nociceptors which are partly upregulated after injury [8]. Unlike peripheral nerves, the spinal cord is protected by the blood-medullary barrier, which was thought to prevent the influx of immune cells into the spinal cord circulation [8]. However, emerging data suggest that peripheral nerve injury may result in disruption of the blood-medullary barrier and allow the influx of peripheral immune cells [8]. Furthermore, there is a central inflammatory response involving cell-type resident in the central nervous system, i.e., microglia and astrocytes. For many years, the role of the central nervous system glia was thought to be limited to neurotrophic support and immune protection [8]. However, it is now established that glia plays an important role in the generation of persistent pain syndromes [8]. Microglial cells are often considered the resident macrophages of the central nervous system due to their shared properties with macrophages. After peripheral nerve injury, the microglial phenotype changes markedly to a pro-inflammatory phenotype in which they proliferate, become highly motile, and phagocytic, expressing new receptors, and releasing pro-inflammatory mediators [8].

## Treatment

The Neuropathic Pain Special Interest Group (NeuPSIG) of IASP underlined the need to create guidelines based on scientific evidence for the pharmacological treatment of NP, taking into consideration the clinical effectiveness, adverse effects, convenience, and costs of each individual drug [17]. Accordingly, different treatment strategies were proposed for the management of NP: these are classified as invasive and non-invasive, pharmacological and non-pharmacological therapies [1]. According to the NeuPSIG guidelines, the use of oral pharmacological therapy as first-line therapy is recommended in patients with NP [17]. Most international guidelines recommend amitriptyline, duloxetine, pregabalin, or gabapentin as first-line agents for the treatment of NP [20]. In particular, gabapentin is approved for the treatment of peripheral NP, while pregabalin is approved for the treatment of peripheral and central NP (Schlereth and Schlereth [18]). Tricyclic antidepressants are used as first-line drugs for the treatment of NP of any etiology [18]). Duloxetine is approved in Germany for the treatment of diabetic neuropathy [18]. Topical therapy is effective in the case of focal nerve injuries with fewer side effects. Topical 5% lidocaine and 8% capsaicin patches are recommended as second-line treatment of localized NP [18]. However, according to Moisset et al. and the “Société française pour l’étude et le traitement de la douleur,” the lidocaine patch can be used as a first-line treatment for peripheral NP [21]. High-concentration topical capsaicin can be used to treat postherpetic neuralgia, HIV neuropathy, and painful diabetic neuropathy [22]. Lidocaine prevents the development of ectopic action potentials by blocking voltage-gated sodium channels, while capsaicin acts as a natural binding of the TRPV1 receptor [18]. Various factors participate in the choice of drug, including possible adverse effects, patient comorbidities, i.e., depression or sleep disorders, possible drug interactions, risk of possible misuse of the drug, abuse, and problems related to costs [17]. However, these guidelines have limitations, as they are not applicable in pediatric age and in patients with trigeminal neuralgia, for whom there are specific recommendations [17]. Sometimes a combination of several drugs may be required to optimize pain control [13]. The association of two or more drugs with different mechanisms of action may provide better pain control on one hand and on the other reduce the adverse effects associated with the use of a single drug at high dose [23].

Add-on therapies, such as physical therapies, exercise training, cognitive behavioral therapy, or psychotherapy may be associated with pharmacological therapy improving results in some patients (Szok [12]). When NP results refractory to drugs, other treatment options have been studied such as peripheral nerve blocks, epidural corticosteroid injection, and Intrathecal drug delivery. Sometimes even these latter approaches may be ineffective or cause side effects [12]. In such cases, the use of techniques of electrical neuromodulation could have an important role. Neurostimulation interrupts or dampens pain signals along neural pathways, either blocking pain transmission or influencing the brain’s processing of pain signals thus reducing pain perception and providing relief to the patient [24]. The stimulation of the motor cortex, vagus nerve, spinal cord, or peripheral nerves is among the most invasive neurostimulation techniques. Among the less invasive techniques, however, there are pulsed radiofrequency therapy, percutaneous or transcutaneous electrical nerve stimulation, transcranial direct current stimulation, and transcranial magnetic stimulation [24].

## Antidepressant drugs

**Fig. 2:**
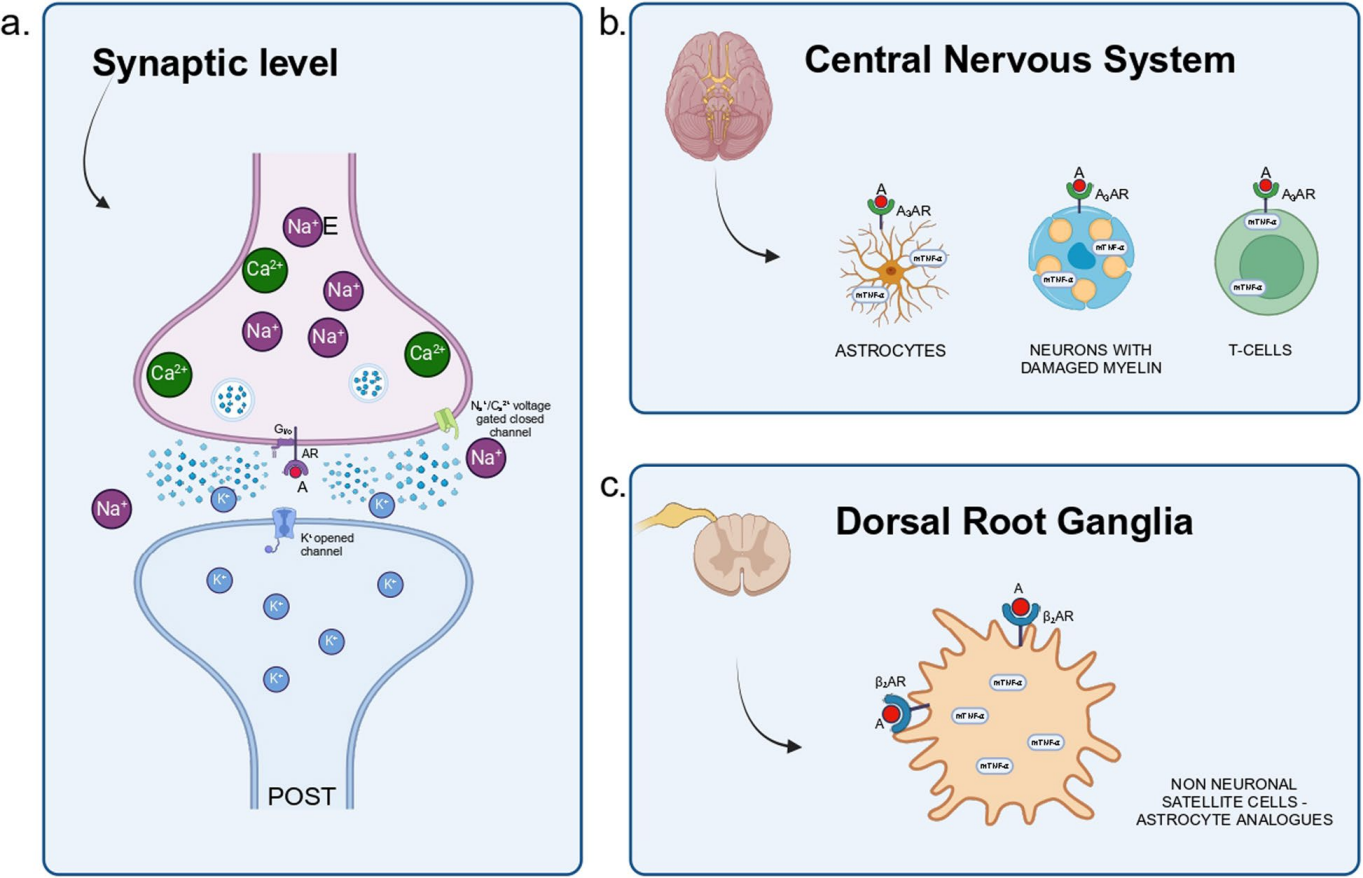
Mechanism of action: antidepressant drugs and modulation of neuroinflammation. a Antidepressants act by binding and inhibiting norepinephrine transporters at the pre-synaptic level, inhibiting the reuptake and therefore causing an increase in norepinephrine levels at the synaptic level. The persistence of norepinephrine in the synaptic cleft determines analgesia due to the presence of α-2-adrenergic receptors, which are coupled to an inhibitory G protein (Gi/o). Its activation determines the inhibition of the Ca 2+ voltage-dependent pre-synaptic channels and therefore inhibition of the release of excitatory neurotransmitters. At the same time, at post-synaptic level, K + channels are opened causing hyperpolarization of the cells and therefore reduction of excitability. This mechanism is of importance against allodynia and hyperalgesia associated with neuropathic pain. Moreover, antidepressants act as sodium channel blockers, inhibiting the ectopic discharges caused by nerve damage. b Amitriptyline plays an important role in the endogenous adenosine system to modulate neuropathic pain. Adenosine receptor A 3 AR is overexpressed in inflammatory cells, such as astrocytes, immune cells, and nociceptive neurons. The activation of A 3 AR by adenosine agonists may mediate antinociception through potentiation of inhibition of GABA, blocking NP. Therefore, amitriptyline is used both because it significantly reduces the expression of the mRNA of pro-inflammatory cytokines and because it determines the activation of the A 3 AR receptor, acting as an adenosine agonist and reducing the inflammatory response. c Nortriptyline has an important antiallodynic effect as it determines the recruitment of norepinephrine in the dorsal root ganglia. It acts on β2-adrenoceptors expressed by non-neuronal satellite cells, which are probably mTNFα-expressing satellite cells. Furthermore, both nortriptyline and venlafaxine inhibit the local production of TNF-α. Blocking TNF-α can relieve symptoms of NP. A, antidepressant drug; AR, adrenergic receptor; A 3 AR, adenosine receptor; Ca 2+ , calcium ion; Gi/o, inhibitory G protein; Na + , sodium ion; K + , potassium ion; POST, post-synaptic level; PRE, pre-synaptic level

According to guidelines, antidepressant drugs are the first-line medications in the management of NP [12]). Antidepressants combine the analgesic action to the management of depression, that is a common comorbidity of patients with NP [17]. The two mechanisms have different timings; the analgesic effects appear within a few days to a week [16], while antidepressant effects are typically evident after 2 or 4 weeks after the first administration [25]). The main mechanism of antidepressants is firstly to increase norepinephrine as neurotransmitter at spinal cord level and secondly to act on the locus coeruleus (LC), thus directly inhibiting pain and activating the impaired descending noradrenergic inhibitory system [25]. Antidepressants act by binding and inhibiting norepinephrine and serotonin transporters at the pre-synaptic level, inhibiting the reuptake of these neurotransmitters and therefore causing an increase in their levels at the synaptic level (Fig. 2) [25]. The persistence of norepinephrine in the synaptic cleft determines analgesia due to the presence of α-2-adrenergic receptors, which are coupled to an inhibitory G protein (Gi/o) [25]. Its activation determines the inhibition of the Ca^2+^ voltage-dependent pre-synaptic channels on the dorsal horn of the spinal cord and therefore inhibition of the release of excitatory neurotransmitters. At the same time, in the post-synaptic level, K^+^channels are opened causing hyperpolarization of the cells and therefore reduction of excitability [25]. This mechanism is of importance against allodynia and hyperalgesia associated with NP [25]. Antidepressants also act on the LC [25]. LC receives inputs from different sites of the central nervous system, regulated by both norepinephrine and serotonin. From LC, descending noradrenergic neurons play a role in an important mechanism of endogenous analgesia. Antidepressants increase norepinephrine, and thanks to α-2-adrenergic receptors, they inhibit the activities of LC [25]. Moreover, antidepressants act as sodium channel blockers, inhibiting the ectopic discharges caused by nerve damage [25]. Some antidepressants act as antagonists of NMDA receptors which are expressed in the neurons of the dorsal horns of the spinal cord, induce wind-up and central sensitization responsible for the onset and maintenance of NP [25]. Tricyclic antidepressants (TCAs) modulate NP on multiple levels: they act on α-1-adrenergic receptors, are calcium channel blockers, potassium channel activators, and adenosine system modulators [25]. Furthermore, TCAs increase the function of GABA-B receptors; they activate opioid receptors and inhibit the production of nitric oxide and prostaglandin E_2_ [25]. Amitriptyline (10–150 mg/day) is one of the first-line drugs indicated by guidelines [17, 18]; its most common side effects are dry mouth, constipation, urinary retention, and orthostatic hypotension [17, 26])due to its anticholinergic action [12]. It is possible to limit these effects, starting with low doses administered before going to bed, and then moving to higher doses only through slow titration. Alternatively, a secondary amine, nortriptyline, can be used [17]. To obtain pain relief, antidepressants should be taken for 6 to 8 weeks, including 2 weeks with the highest tolerated dosage [17]. More recently, selective serotonin and norepinephrine reuptake inhibitors (SSNRI) have been introduced into the guidelines: they are better tolerated but they are less effective than tricyclic antidepressants [17]). Venlafaxine (150–225 mg/day to be administered once a day) and duloxetine (60–120 mg/day to be administered once a day) are commonly prescribed. The most frequent side effect of duloxetine is nausea [26], which can be limited by starting at a dosage of 30 mg once a day for 1 week and then increasing to 60 mg [17]. Instead, venlafaxine may cause cardiac conduction anomalies or increased blood pressure [17]. Furthermore, venlafaxine necessitates a decalage of the dosage due to possible withdrawal syndrome [17].

## Antidepressants and neuroinflammation

Antidepressants are not primally analgesic drugs; however, they are used in the treatment of NP because they recruit long-term neuronal plasticity [27]. Kim et al. have investigated the antinociceptive effect of antidepressants and specifically of amitriptyline, explaining the role of the endogenous adenosine system in NP in a rat model [7]. Adenosine receptor A_3_AR is overexpressed in inflammatory cells, such as astrocytes, immune cells, and nociceptive neurons [7]. It modulates inflammation and apoptosis in many organs. Liao et al. showed that autophagy can help in the removal of myelin and promote nerve regeneration reducing NP [10]. However, there is no direct evidence to show that inhibiting apoptosis in damaged neurons can attenuate pain [10]. The activation of A_3_AR by endogenous adenosine or adenosine agonists may mediate antinociception through potentiation of inhibition of GABA, blocking NP [7]. Therefore, amitriptyline is used both because it significantly reduces the expression of the mRNA of pro-inflammatory cytokines and because it determines the activation of the A_3_AR receptor, acting as an adenosine agonist and reducing the inflammatory response (Fig. 2) [7].

García-Fernandez et al. proved that the local inflammatory process could be triggered by the activation of pathways mediated by toll-like receptors (TLRs) [28]. TRL-4 receptor determines the release of pro-inflammatory cytokines such as TNF-α through the upregulation of a cascade mediated by NFkB. TNF-α is a pro-inflammatory cytokine that drives and stimulates the hyperinflammation in NP [9]. It also controls two programmed mechanisms of cell death, apoptosis, and necroptosis [9]. TNF-α produced at the peripheral level could be transported to the central nervous system level via axonal transport: this is why the literature strongly supports the role of TNF-α in the pathogenesis of NP [5].

Bohren et al. studied the relationship between allodynia in NP and the role of antidepressants on a mice model [6]. Norepinephrine acts selectively on β2-adrenoceptors, so repeated interaction of these receptors with direct agonists is sufficient to achieve a therapeutic effect. In neuropathy, norepinephrine can be released within supraspinal structures, spinally via descending noradrenergic pathways, and peripherally in the dorsal root ganglia. However, an important target in NP might be located at the spinal cord and/or dorsal root ganglia, rather than at the supraspinal level [6]. Nortriptyline has an important antiallodynic effect as it determines the recruitment of norepinephrine in the dorsal root ganglia [6]. It acts on β2-adrenoceptors expressed by non-neuronal satellite cells, which are probably mTNFα-expressing satellite cells (Fig. 2) [6]. These cells can be considered the peripheral analogues of astrocytes of the central nervous system. Furthermore, both nortriptyline and venlafaxine inhibit the local production of TNF-α. Following nerve injury in the dorsal root ganglia of the peripheral nervous system or in the spinal cord or supraspinal structures of the central nervous system, glial and immune cells induce the production of pro-inflammatory cytokines such as TNF-α, which contribute to the onset of NP [6]. Blocking TNF-α can relieve symptoms of NP [6]. Antidepressant drugs would act as indirect anti-TNF-α drugs to relieve neuropathic allodynia. This direct anti-TNF-α effect is partial as it suppresses neuropathy-induced TNF-α overexpression without affecting the basal expression of the cytokine [6]. Sud et al. examined the relationship between the production of TNF-α and the activation of the α-2-adrenergic receptor, since TNF-α regulates the activity of noradrenergic neurons in the brain, which are the ones that direct the descending modulation of pain [5]. The activation of the α-2 adrenergic receptor can increase or inhibit TNF-α production depending on extracellular levels of TNF-α [4]. TNF-α regulates the coupling of the α-2-adrenergic receptor with specific G proteins, thus directing the release of the neurotransmitter and the signal transduction of the same receptor [5]. Alteration of the balance, caused by a pathological increase in TNF-α, could cause a dysfunctional adaptation of the α-2 adrenergic receptor with the development of chronic pain [5]. Amitriptyline probably disturbs this balance between TNF-α production and activation of the α-2 adrenoreceptor [5]. Specifically, following the administration of antidepressants, there is a reduction in the production of TNF-α in the brain which in turn transforms the function of the α-2-adrenergic receptor from an inhibitor to an activator of the release of norepinephrine [5]. This transformation is fundamental because following the development of chronic pain, inhibition of the α-2-adrenergic receptor develops and therefore leads to a reduced release of norepinephrine [5]. A summary of the relevant studies on the topic is displayed in Table 1.

**Table 1:** Literature analyzing the relationship between neuropathic pain, neuroinflammation and antidepressant agents*.*

Author, year doi	Title	Study design	Aim	Findings
(Fang et al. [31]) 10.9758/cpn.2019.17.2.189	Abnormalities in Inflammatory Cytokines Confer Susceptible to Chronic Neuropathic Pain-related Anhedonia in a Rat Model of Spared Nerve Injury	Interventional study on experimental model of rats	To investigate the role of inflammatory cytokines in NP-related anhedonia and to explore the effects of ketamine and parecoxib on pain and anhedonia	TNF-α and IL-1β are increased in the medial prefrontal cortex, serum, and spinal cord of anhedonia-susceptible rats. Anhedonia-unsusceptible rats increased the interleukin IL-1β level in the medial prefrontal cortex, serum, and spinal cord. IL-6 was altered in serum and spinal cord, but not in the medial prefrontal cortex. IL-10 was significantly altered in the medial prefrontal cortex and serum, but not in the spinal cord. Additionally, ketamine treatment significantly attenuated the decreased results of mechanical withdrawal test and sucrose preference test in anhedonia-susceptible rats, and that parecoxib significantly improved the mechanical withdrawal test score, but failed to alter the result of sucrose preference test
Liao et al. [10] 10.3390/ijms23052685	The Role of Autophagy and Apoptosis in Neuropathic Pain Formation	Review	To summarize the complex protein and molecular modifications of the autophagy process in NP and the alteration of pain behaviors after modulating autophagic/apoptotic activities. To discuss autophagic/apoptotic activity changes and their interactions with pro-inflammatory cytokines based on the different anatomical locations	Autophagic and apoptotic activities in injured nerves and dorsal root ganglia increase after nerve damage. The upregulated autophagic activities could possibly help myelin clearance and promote nerve regeneration, which can attenuate pain behavior. The interaction of autophagy and apoptosis in spinal cord NP formation mainly involves the modulation of pro-inflammatory cytokines. Alterations in autophagic/apoptotic and pro-inflammatory cytokine activities over time in the spinal dorsal horn play essential roles in NP formation after nerve damage
Duan et al., [9] 10.3390/ijms23137191	Neuroimmune Mechanisms Underlying Neuropathic Pain: The Potential Role of TNF-α-Necroptosis Pathway	Review	To analyze TNF-α pathway in pain, to summarize studies related to the mechanisms of NP mediated by TNF-α, and to discuss the role of the TNF-α–necroptosis pathway	TNF-α can regulate cation channels to sensitize primary afferents in the peripheral nervous system, affect excitatory and inhibitory synaptic transmissions in central nervous system, and evoke positive feedback between TNF-α and microglial activation to induce neuroinflammation, thus facilitating pain transmission, adverse pain-associated emotional reactions and cognitive deficits. TNF-α-triggered necroptosis, a novel form of programmed cell death, may be one of the key factors for inducing neuroimmune responses in NP
(Kim et al. [7]) 10.4097/kja.d.18.00022	Amitriptyline inhibits the MAPK/ERK and CREB pathways and pro-inflammatory cytokines through A3AR activation in rat neuropathic pain models	Interventional study on experimental model of rats	To investigate whether amitriptyline is effective in relieving the symptoms of NP and whether the effect of amitriptyline was associated with endogenous adenosine, particularly A3AR	The level of phospho-ERK1/2 and phospho-CREB proteins and pro-inflammatory cytokines are reduced by amitriptyline administration. However, the use of MRS-1191 before amitriptyline administration increased the signaling protein and pro-inflammatory cytokine levels, which were reduced by amitriptyline
(Bohren et al. [6]) 10.1016/j.nbd.2013.08.012	Antidepressants suppress neuropathic pain by a peripheral β2-adrenoceptor mediated anti-TNFα mechanism	Interventional study on experimental model of mice	To demonstrate that the peripheral nervous system is essential for the antiallodynic effect of nortriptyline	By recruiting noradrenaline from sympathetic fibers sprouting in the dorsal root ganglia, antidepressant drugs stimulate local β2-ARs on non-neuronal cells. This action decreases mTNFα production and leads to the antiallodynic effect
Zhang et al., [29] 10.1111/cns.13807	Inhibition of phosphodiesterase-4 in the spinal dorsal horn ameliorates neuropathic pain via cAMP-cytokine-Cx43 signaling in mice	Interventional study on experimental model of mice	To determine the effect of phosphodiesterase-4 inhibitors rolipram and roflumilast on partial sciatic nerve ligation-induced mechanical hypersensitivity. To observe the role of cAMP-Cx43 signaling in the effect of phosphodiesterase-4 inhibitors on partial sciatic nerve ligation-induced mechanical hypersensitivity	Single or repeated, intraperitoneal or intrathecal administration of rolipram or roflumilast reduced mechanical hypersensitivity following partial sciatic nerve ligation. In addition, repeated intrathecal treatment with either of phosphodiesterase-4 inhibitors reduced PSNL-induced downregulation of cAMP and Cx43, and upregulation of pro-inflammatory cytokines TNF-α and IL-1β. Furthermore, the antinociceptive effects of phosphodiesterase-4 inhibitors were attenuated by the protein kinase A (PKA) inhibitor H89, TNF-α, or Cx43 antagonist carbenoxolone. Finally, partial sciatic nerve ligation induced upregulation of phosphodiesterase-4B and phosphodiesterase-4D, especially the phosphodiesterase-4B subtype, was reduced by treatment with either of the phosphodiesterase-4 inhibitors
Royds et al., [30] 10.1016/j.euroneuro.2019.12.106	An investigation into the modulation of T cell phenotypes by amitriptyline and nortriptyline	In vitro study on peripheral blood mononuclear cells	To examine the effects of amitriptyline on T cell activation and cytokine production	Amitriptyline modulates immune function via T cell and cytokine regulatory pathways. amitriptyline can attenuate the T_H_1/T_H_17 immune response and modify immunomodulatory pathways in vitro

*A3AR A3* adenosine receptor, *CREB* cyclic *AMP* response element-binding protein, *ERK* extracellular signal-regulated kinase, *MAPK* mitogen-activated protein kinase, MRS* 3*-ethyl-5-benzyl- 2-methyl-4-phenylethynyl-6-phenyl-1,4-( ±)-dihydropyridine-3,5-dicarboxylat

The potential role of antidepressants in modulating neuroinflammatory pathways involved in NP has been the subject of preclinical studies in vitro and on animal models, strengthening the basis of their use in the pharmacological treatment of NP [6, 7, 29–31]. On the other hand, clinical research on human patients did not investigate this specific mechanism, focusing instead on drug vs placebo studies which assessed the efficacy of antidepressants as first-line treatment for several cases of neuropathic pain [3].

## Conclusion

Neuropathic pain is a chronic condition, which is difficult to diagnose and to treat. Neuroinflammation plays a fundamental role in the onset and modulation of neuropathic pain. During neuropathic pain, inflammatory cytokines and neurotransmitters are the main players in a bidirectional communication between the central and peripheral nervous system and the immune system. The modulation of these mediators could have important implications on the management of neuropathic pain. Antidepressants seem to act on mediators of neuroinflammation reducing or abolishing the symptoms related to neuropathic pain helping patients’ quality of life improvement. Increasing knowledge on the interaction between neuroinflammation and the use of antidepressants could lead to a more thorough understanding of mechanisms, helping to achieve an optimal management of patients with Neuropathic pain and their symptoms.

## Data Availability

No datasets were generated or analysed during the current study.

## Abbreviations

DN4: Douleur Neuropathique en 4 Questions
IASP: International Association for the Study of Pain
LANSS: Leeds Assessment of Neuropathic Symptoms and Signs
LC: Locus coeruleus
NaV: Voltage-gated sodium channels
NeuPSIG: Neuropathic Pain Special Interest Group
NP: Neuropathic pain
NPS: Neuropathic Pain Scale
NPSI: Neuropathic Pain Symptom Inventory
NRS: Numeric rating scale
QST: Quantitative sensory testing
TCAs: Tricyclic antidepressants
TLR: Toll-like receptor
TNF-α: Tumor necrosis factor-alpha
TRP: Thermotransient receptors
